# Perivascular Spaces, Glymphatic System and MR

**DOI:** 10.3389/fneur.2022.844938

**Published:** 2022-05-03

**Authors:** Linya Yu, Xiaofei Hu, Haitao Li, Yilei Zhao

**Affiliations:** ^1^Department of Radiology, The First Affiliated Hospital, School of Medicine, Zhejiang University, Hangzhou, China; ^2^Department of Radiology, Southwest Hospital, Third Military Medical University (Army Medical University), Chongqing, China

**Keywords:** perivascular space (PVS), CSVD, glymphatic system, magnetic resonance imaging, aquaporin 4 (AQP4)

## Abstract

The importance of the perivascular space (PVS) as one of the imaging markers of cerebral small vessel disease (CSVD) has been widely appreciated by the neuroradiologists. The PVS surrounds the small blood vessels in the brain and has a signal consistent with the cerebrospinal fluid (CSF) on MR. In a variety of physio-pathological statuses, the PVS may expand. The discovery of the cerebral glymphatic system has provided a revolutionary perspective to elucidate its pathophysiological mechanisms. Research on the function and pathogenesis of this system has become a prevalent topic among neuroradiologists. It is now believed that this system carries out the similar functions as the lymphatic system in other parts of the body and plays an important role in the removal of metabolic waste and the maintenance of homeostatic fluid circulation in the brain. In this article, we will briefly describe the composition of the cerebral glymphatic system, the influencing factors, the MR manifestations of the PVS and the related imaging technological advances. The aim of this research is to provide a reference for future clinical studies of the PVS and glymphatic system.

## Introduction

Approximately 150 years ago, German pathologist Rudolf Virchow (1821–1902) and French anatomist Charles Philippe Robin (1821–1885) described the perivascular space in detail. As a tribute to them, perivascular space was named Virchow-Robin space (VRS). A VR space is a space filled with interstitial fluid (ISF) surrounding the cerebral small vessels (CSV). They penetrate the brain parenchyma from the brain surface, and are covered with leptomeninges. PVS is an emerging and early imaging marker of CSVD and can be considered a risk factor for CSVD. The STandards for ReportIng Vascular changes on nEuroimaging (STRIVE) (1) lists PVS together with recent small subcortical infarct (RSSI), lacunae, white matter hyperintensity (WMH), cerebral microbleed (CMB) and cerebral atrophy as the six major imaging manifestations of CSVD. Besides CSVD, many neurodegenerative disorders are associated with dilated PVS, such as Alzheimer's Disease (AD), Parkinson's Disease (PD), multiple sclerosis (MS), idiopathic normal pressure hydrocephalus (iNPH), brain trauma, central nervous system (CNS) cryptococcal infection and mucopolysaccharidosis.

While the function of PVS has been debated, the current literature consensus suggests that PVS acts as a conduit for lymphatic drainage, exchange between CSF and ISF, and waste removal from the brain, and it is the anatomic basis of the glymphatic system (2).

In the following review, we provide a brief description of the glymphatic system in terms of its function and possible mechanisms. We also discuss MR imaging features of VRS. In addition, we focus on the novel MR imaging techniques for evaluating the glymphatic system, some of which are currently in development but likely to become available soon in clinical settings.

## Anatomy of PVS

**Figure 1:**
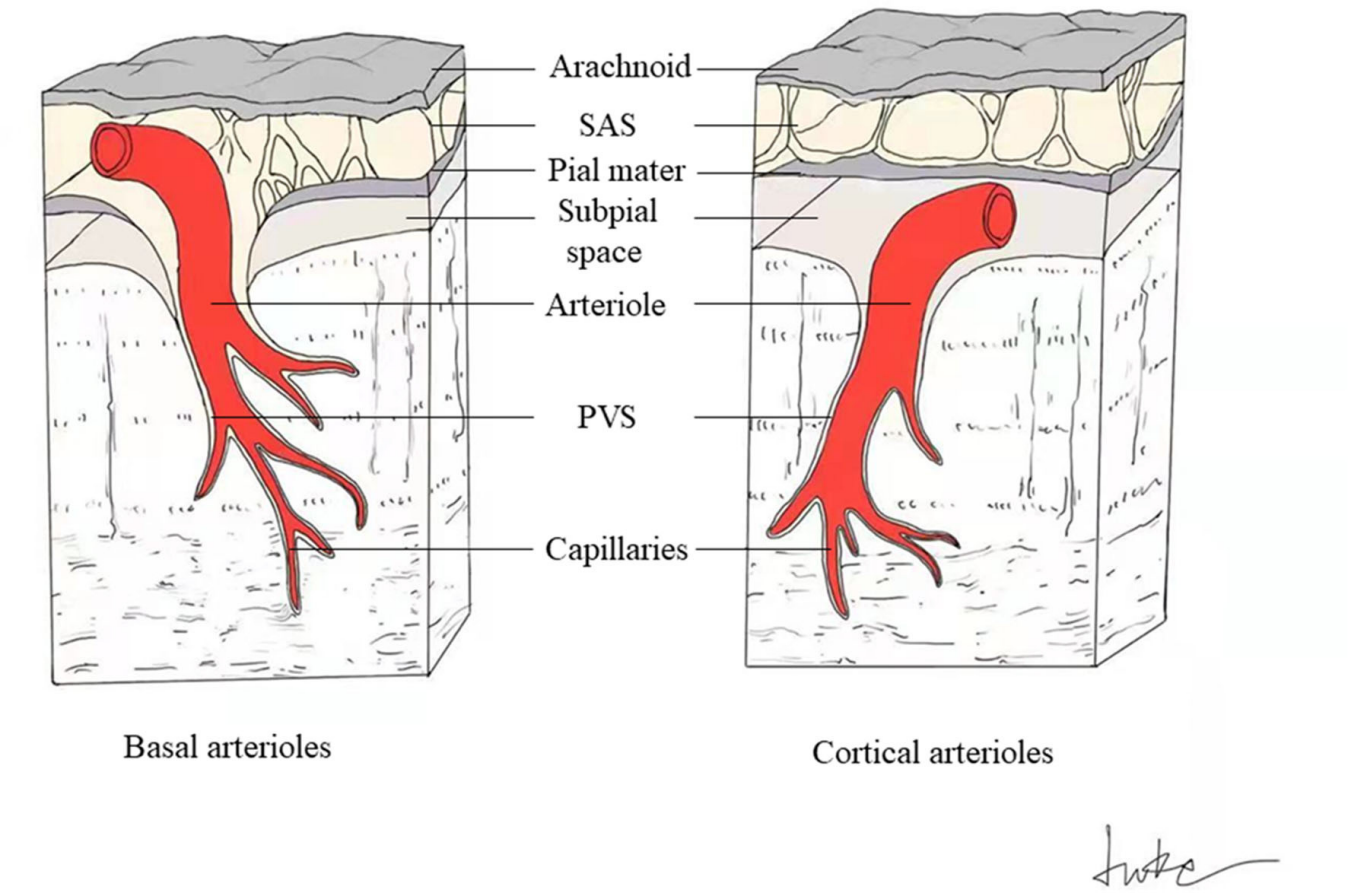
Schematic drawing of PVS around basal and cortical perforating arterioles. The perivascular spaces surround the cerebral small perforating vessels as they pass through the brain parenchyma from the subarachnoid space (SAS). The outer limits of PVS are the glia limitans of the underlying brain, and inner limits are the endothelial basement membranes of the vessel. The basal perforating arterioles are covered by two layers of leptomeninges, with the inner membrane tightly adhering to the arteriolar wall, and the outer layer extending from the pia mater (left). The spaces between these two meningeal membranes connect to the SAS directly (left). Cortical arterioles are surrounded by only one leptomeningeal layer, closely applied to the vessel wall, so the cortical perivascular spaces connect directly to the subpial space and indirectly to the SAS in some way (right).

VR spaces surround the walls of arteries, arterioles, capillaries, veins, and venules as they pass through the brain parenchyma from the subarachnoid space. The outer limits of VR spaces are the glia limitans of the underlying brain, and inner limits are the endothelial basement membranes of the vessel (Figure 1) (3). Penetrating arterioles are completely wrapped in a sheet of pia mater. It can be observed under electron microscopy that when the penetrating arterioles branch into the level of capillaries, the basement membrane of the pial sheath and the basement membranes of the astrocytes (glia limitans) fuse together to create a perivascular compartment (4). The compartment separates the vessel from surrounding brain tissue and fill with ISF.

The perforating arteries in the basal ganglia (BG) are covered with two coats of leptomeninges, and the spaces between these two layers connect to the subarachnoid space directly (Figure 1) (3). Cortical arterioles and all venules are surrounded by only one leptomeningeal layer, so they connect directly to the subpial space and indirectly to the subarachnoid space in some way (Figure 1) (3).

VR spaces are accessory structures that are not actually a part of the blood vessel. It has been demonstrated that VR spaces function as pathways for waste removal and fluid drainage in the brain, similarly to the lymphatic drainage system in the body (2, 5).

## Glymphatic System and PVS

### Glymphatic System Hypothesis

Although the existence of perivascular spaces has been confirmed by pathologists for more than 150 years and MR has made it possible to clearly visualize enlarged perivascular spaces (EPVS), the function and clinical significance of perivascular spaces continued to be debated until 2012, when Iliff et al. (5) uncovered a new mechanism of brain metabolism-a unique para-vascular pathway of brain metabolism, thus gradually unraveling the mystery of perivascular spaces. Their study revealed the presence of interstitial solute clearance structures dependent on astrocyte podocyte aquaporins 4 (AQP4) in the perivascular space of the rodent brain and termed it the “glymphatic system” (5). Iliff et al. also found that soluble amyloid β (Aβ) protein injected into the striatum of mice was cleared from this para-vascular pathway (5). In their subsequent study, the exchange of CSF with ISF in the live rat brain was observed by dynamic contrast-enhanced MRI through intrathecal injection of two different molecular weights of MRI contrast agents. Both contrast agents passed largely similarly through the para-arterial space in the rats, but the smaller molecular weight contrast agent entered the brain parenchyma more extensively, while the larger one was sparsely distributed in the brain parenchyma (6). This finding points to the molecular size-selective nature of the glymphatic system for clearing substances, which may explain the tendency of large molecular Aβ protein to be deposited around blood vessels. The discovery of meningeal lymphatic vessels in 2015 was even stronger evidence for the existence of brain glymphatic system (7).

**Figure 2:**
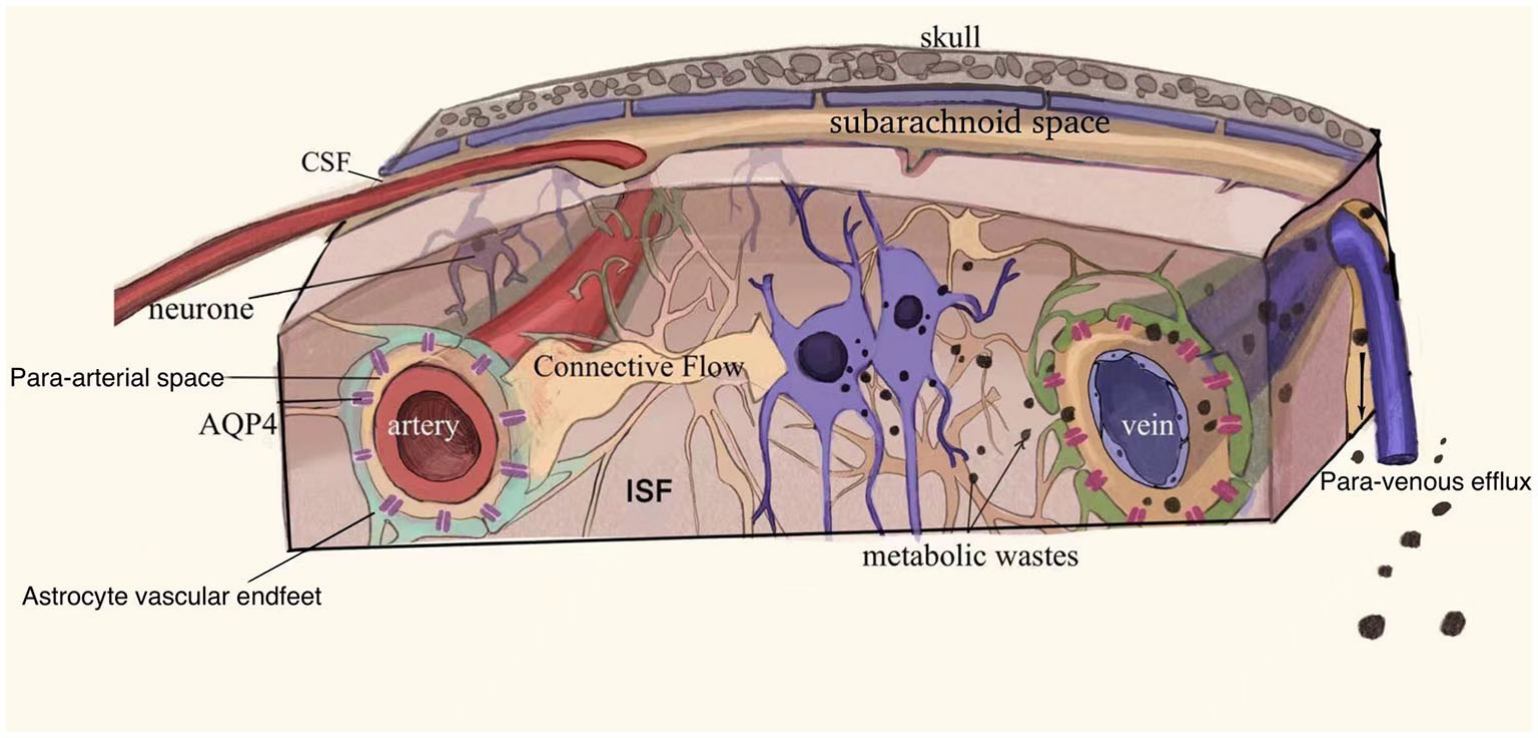
Schematic drawing of the glymphatic system. The glymphatic system may consist of the following three principal segments (1) a para-arterial CSF influx, (2) an astrocyte end-foot water channel protein AQP4 mediated convection of CSF and ISF, and (3) a para-venous ISF efflux. Neuronal metabolites are carried into the perivascular space and drain through the meningeal lymphatic vessels and soft tissues surrounding the skull to the cervical and nasal lymph nodes and are eventually removed from the general circulation. Adapted with permission from Nedergaard (2).

The precise drainage pathways and mechanisms of the glymphatic system are not known. It is hypothesized that the system consists of three principal segments (2, 8): (1) a para-arterial CSF influx, (2) an astrocyte end-foot water channel protein AQP4 mediated convection of CSF and ISF, and (3) a para-venous ISF efflux (Figure 2). The possible circulation pathways of the system are as follows: neuronal metabolites are carried to the perivascular space via convection of CSF and ISF facilitated by AQP4, from where they are transported directed into lymphatic vessels located in meninges and in the soft tissue surrounding the skull and eventually return to the general circulation for clearance by the kidneys and liver via cervical and nasal lymph nodes (Figure 2) (2, 7, 8).

### Drivers of Glymphatic Transport

The possible factors driving ISF through perivascular spaces are continuous production of CSF (8), vascular pulsation (9, 10), respiratory movement (11), the sleep–wake cycle (12–14), and AQP4 water channel protein (15).

The power source for this fluid drainage system is generated by the continuous CSF flow. The production of CSF from the choroid plexus drives the fluid from the ventricular system into the subarachnoid space and along the perivascular space into the cerebral interstitium. The driving force generated by CSF flow is important for the exchange between CSF and ISF and helps to flush out metabolites within perivascular spaces (8).

Cerebrovascular pulsatility drives fluid transport along this pathway. Rodent studies have shown that increased intracranial vascular pulsation is associated with increased PVS, and that vascular pulsation, particularly in the small cerebral arteries, is directly involved in the production of enlargement of perivascular spaces in the brain (9, 10). The elevation of blood pressure and the increase in the amplitude of pulsation of the arterial wall, although the arterial diameter remains constant, causes a decrease in the net flow of PVS and a decrease in debris clearance (10). Spontaneous low-frequency oscillations were observed in the arteries of awake mice which drives the drainage of solutes from the PVS (16). In mice with cerebral amyloid angiopathy (CAA), impaired vascular reactivity resulted in reduced solute clearance (16).

The similar effect of vascular pulsation on the glymphatic function of the human brain is derived from phase contrast MR. Lower cerebrovascular reactivity (CVR) was associated with higher WMH volume and increased PVS visibility in the basal ganglia (17). Furthermore, increased intracranial vascular pulsatility was associated with lower CSF stroke volume at the foramen magnum and, in turn, with increased PVS burden at the basal ganglia (17).

Respiratory movements affect the fluid drainage within the PVS by influencing CSF transport. Forced inspiration causes the CSF to move cephalad, while the venous blood draws away caudally (11). The movement of CSF from breathing might also flush perivascular spaces.

The glymphatic system is more effective during sleep. Using real-time assessment of tetramethylammonium diffusion and two-photon imaging, a recent study evaluated tracer clearance in live mice during wakefulness, natural sleep, and anesthesia. They found that the rate of clearance was highest during sleep, with the same performance under anesthesia, and a 95% reduction in tracer flux into the perivascular spaces of the mouse brain during wakefulness (12). Similarly, the clearance of Aβ by mice doubled during sleep (12). The study also shows a 60% increase in interstitial space under natural sleep or anesthesia, thus explaining the significant increase in the convection between CSF and ISF. Sleep may be involved in perivascular clearance in ischemic encephalopathy (13). CSF uptake and ISF drainage from the PVS increases during sleep, and poor sleep quality may affect the clearance of neurotoxins, interrupting ISF drainage and possibly leading to enlarged perivascular spaces (13). Hablitz et al. studied the relationship between the sleep timing and the glymphatic function in mice and found that the influx and clearance of glymphatic fluid had a circadian rhythm and peaked in efficiency during the mid-rest phase (14). The peak of perivascular AQP4 polarization occured simultaneously during the resting phase in mice, and the absence of AQP4 eliminated diurnal differences in glymphatic flow and drainage (14). Therefore, they concluded that the distribution of CSF is controlled by the diurnal rhythm, and AQP4 is involved.

AQP4 is a water channel protein on the feet of perivascular astrocytes and is a regulator of normal glymphatic function (15, 18), which facilitates the exchange of CSF and ISF in the VR spaces and the active outflow of ISF (5). Although AQP4 is the major water channel protein in mammals, it is only expressed in astrocytes and ventricular meningeal cells. The outer wall of the VR space is composed of astrocyte end-feet with abundant AQP4, which allows water in this interstitial space to move into the ISF, making the transport of small molecules and ions possible. In AQP4 knockout mice and rats, the transport of tracers in the CSF and ISF was significantly reduced, suggesting that the AQP4 gene is necessary for the fluid transport in the brain (5, 15). But the importance of AQP4 in the glymphatic system remains controversial. Smith et al. founded that uptake of CSF tracers and transport of fluorescent dextrans from subarachnoid space to brain parenchyma were unaffected by AQP4 gene knockout in rats or mices (19). They suggested that AQP4 does not appear to be necessary for fluid movement within the glymphatic system in rodent brains, and this movement applies more to diffusion than to convection. These conflicting findings may be due to the use of different techniques or anesthetics by different investigators (3, 20).

### Substances Cleared by the Glymphatic System

Glymphatic clearance is a molecular size selective mechanism (5, 6). Soluble Aβ (5), tau (3) and lactate (21) can be selectively removed through this system. The system also contributes to the delivery of apolipoprotein E (ApoE) (22), lipids (23) and signaling molecules (23). AQP4 plays a major role in the transport of ions and small molecules. Polarized AQP4 can fold toward the center and constitute highly selective water pores, allowing the smooth passage of water molecules (2, 5, 6, 8). The abundance of AQP4 on the astrocyte end-foot, which forms the outer wall of the perivascular space, provides for the transport of small molecules and ions. At the same time, the cleft between astrocyte end-feet allows the passage of macromolecular compounds, and most of the solutes with molecular weight <100 kDa will leave the perivascular spaces through this cleft (8). Today's research is increasingly focused on the glymphatic clearance mechanisms, hoping to develop new therapeutic strategies for associated neurological disorders in the future.

### PVS Enlargement

The PVS, as the main conduit for the drainage of ISF from brain tissues, is responsible for the exchange between CSF and ISF, the removal of intracerebral waste, and the maintenance of brain homeostasis (2). PVS also acts as a conduit for delivering various signaling molecules and metabolic factors (2, 18). The precise causes and mechanisms of PVS enlargement are not yet known. As the anatomical basis of the glymphatic system, the increased visibility and enlargement of PVS on imaging may be potentially linked to the dysfunction of the glymphatic system. Theoretically, PVS enlargement should be caused by fluid accumulation due to imbalance between CSF inflow and outflow, but direct evidence is still lacking (24). Altered blood flow and blood-brain barrier (BBB) dysfunction are also believed to be involved in PVS dysfunction (24, 25).

It is controversial whether the visible PVS on MRI refers only to the periarterial spaces or to both the periarterial and perivenous spaces. Most of the evidence suggests that visible PVS on MR are around arteries (3). However, the answer to the question of whether the absence of visibility on MR equates to the absence of enlargement of the perivenous spaces or the absence of venular dysfunction is no. Histopathological studies of autopsy specimens confirm the presence of many visible perivenous spaces in areas of collagenization of deep penetrating veins (3, 26). However, the extent to which this venous collagenization contributes to the visibility of PVS on MR, and the incidence and severity of venous collagenization in small vessel disease are unknown. Furthermore, the available studies do not find that the main pathway of solute efflux in the glymphatic system is along perivenous routes, but rather there is much evidence that solute inflow and efflux occur along periarterial routes (27). If the main pathway of solute efflux is not perivenous routes, could this explain to some extent why enlarged perivenous spaces are usually not observed on MR.

Patients with dilated VRS are generally asymptomatic, but multiple enlarged VRS or giant VRS may occasionally show the following symptoms or signs: headache, dizziness, cognitive impairment, inattention, visual impairment, epilepsy. PVS may be aggravated by aging, hypertension, inflammation, lacunar stroke, and dementia (3).

The enlarged perivascular space (EPVS) at different sites may have different pathophysiological basis and pathogenic mechanisms. EPVS often coexists with lacunar infarction. EPVS tends to be more pronounced in the basal ganglia region in patients with lacunar stroke than in those with non-lacunar stroke (28). Ischemic stroke patients with a higher EPVS load in the basal ganglia region have a higher risk of stroke recurrence (24). Small artery occlusion stroke subtypes have a relatively higher risk of high load EPVS in the hippocampus (29). Patients with CAA have a higher total cortical Aβ load and impaired Aβ clearance leading to enlarged perivascular spaces, especially in the centrum semiovale (30, 31). In patients with spontaneous cerebral hemorrhage, basal ganglia EPVS severity is associated with hypertensive arteriopathy (31), and high EPVS load in the centrum semiovale is associated with cerebral hemorrhage recurrence in patients with CAA (32). Post-stroke depression (PSD) is a common psychiatric symptom after stroke, occurring in approximately 1/3 of patients (33). EPVS is thought to be associated with the development of PSD. Researchers found that centrum semiovale rather than basal ganglia EPVS is more severe in patients with PSD and is associated with poor antidepressant response (34).

There are conflicting data on MR regarding the relationship between perivascular spaces and cognitive decline. Most studies have concluded that the burden of EPVS in both the basal ganglia and centrum semiovale is associated with cognitive decline (3). However, some studies did not find an association between EPVS in the basal ganglia region and cognitive dysfunction (3), whereas Arba et al. came to the opposite conclusion, suggesting that EPVS in the basal ganglia region, but not in the centrum semiovale, was associated with cognitive decline (35). Three other studies showed no independent association between EPVS and cognitive impairment (36, 37) and that EPVS was not a predictor of cognitive decline (38). These contradictory data may be related to the differences in the enrollment and assessment methods of each study. In addition, due to the wide range of cognitive functions included, EPVS may affect only some of these cognitive domains and not necessarily the overall cognitive function of the whole brain.

In addition, CADASIL 45, PD, cerebral trauma, idiopathic normal pressure hydrocephalus, mucopolysaccharidosis (39), neuropsychiatric retardation, autism (40), systemic lupus erythematosus (SLE) (41, 42), multiple sclerosis, primary angiitis of the central nervous system (43, 44), and cryptococcal infection (45, 46) are often accompanied with EPVS. For example, in patients with mucopolysaccharidosis, transient sieve-like PVS can appear in the white matter in the early stages of the disease and can increase, decrease or remain stable depending on the progression of the disease (39). In patients with cryptococcal infection, multiple EPVS can be found in the cerebral hemispheres, parietal ventricles, midbrain, bilateral cerebellar hemispheres, and especially in the basal ganglia, which is considered the most common early sign of cryptococcal infection and is associated with intracranial dissemination and proliferation of cryptococci (45, 46).

EPVS is not a specific disease, but should be considered as one of the imaging markers for the development of multiple diseases, and its mechanism of action and corresponding clinical relevance in neurological diseases are still unclear and need further investigation.

## MR Imaging of Perivascular Spaces

### Appropriate MR Imaging Sequence

Based on STRIVE (1), PVS were referred to as a neuroimaging marker of CSVD. MRI instead of CT is recommended for detecting PVS. The recommended sequences include T1-weighted imaging (T1WI), T2-weighted imaging (T2WI), fluid attenuated inversion recovery sequence (FLAIR), diffusion-weighted imaging (DWI) and apparent diffusion coefficient map (ADC). VRS are best detected using T2WI. DWI is the most sensitive sequence for RSSI. FLAIR is used to identify VRS from WMHs and lacunes. A field strength of 1.5T is often of a similarly high resolution as 3.0T (47). It is generally believed that the VRS seen on conventional magnetic field strength (1.5T or 3.0T) MR is the peri-arteriolar space (48).

### MR Manifestation

The signal intensity of perivascular spaces is consistent with CSF on all MRI sequences showing hypointense on T1WI and FLAIR, hyperintense on T2WI, without enhancement and generally no mass effect (1, 49). The diffusion of PVS is unrestricted. The signal of the surrounding brain is generally normal.

**Figure 3:**
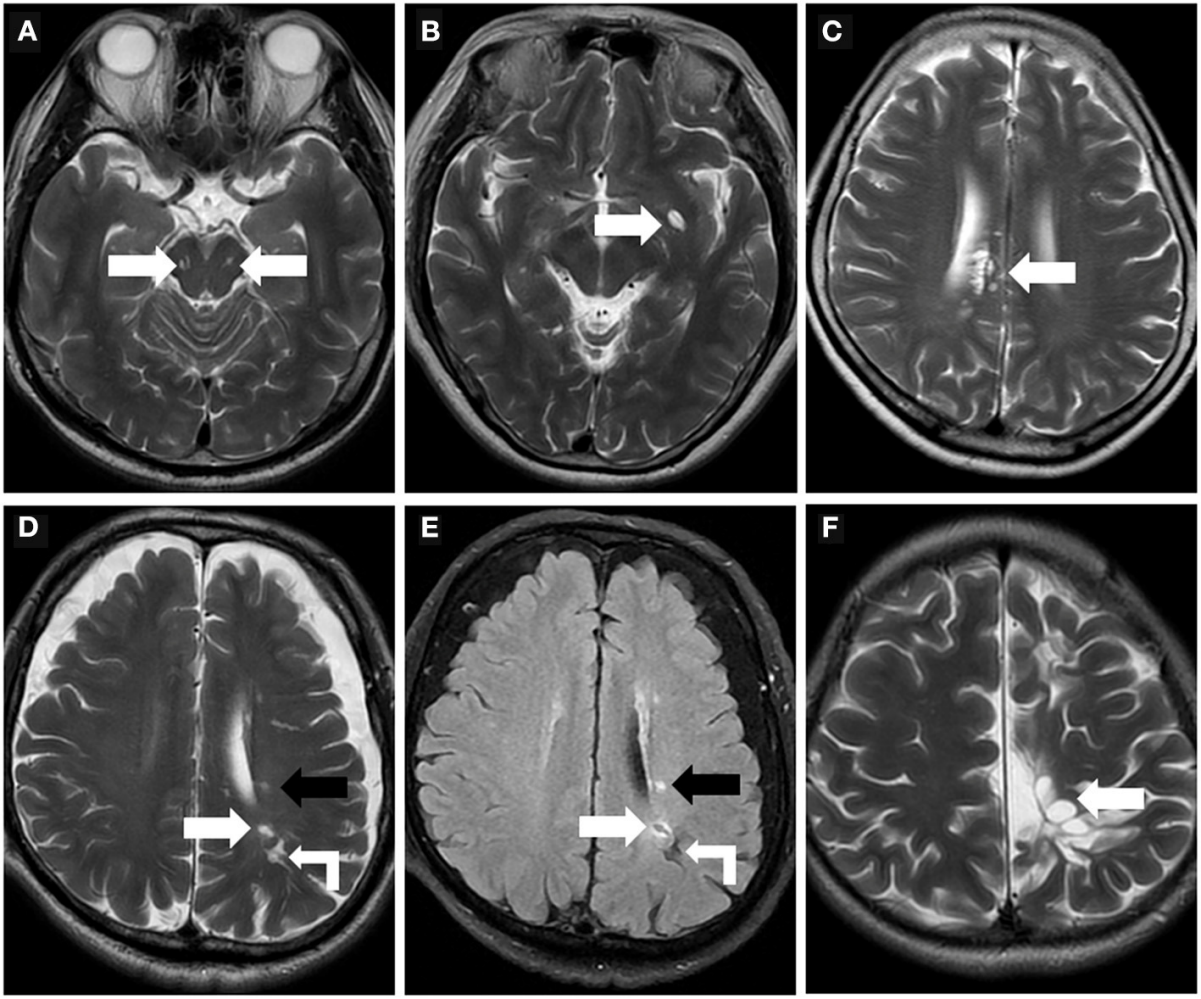
Morphologic characteristics of the perivascular spaces. **(A)** Type III-midbrain type: Axial T2WI showed multiple linear EPVS (white arrow) with high intensity in the midbrain, which were generally symmetrically distributed on both sides and consistent with the course of the penetrating arteries. **(B)** In a 62-year-old woman, axial T2WI showed a large EPVS in the left precribrum with sac-like high intensity, within which a linear hypointense vessel can be seen, namely “vessel sign” (white arrow). **(C)** Axial T2WI revealed multiple linear and ovoid EPVS clustered locally near the right lateral ventricle (white arrow). **(D,E)** In a 76-year-old man with a history of hypertension and diabetes mellitus for many years, EPVS (white right angle arrow), WMH (black arrow), and small lacune (white arrow) were simultaneously present in the left centrum semiovale. The lacune (white arrow) showed a hyperintense rim around the vesicular cavity on FLAIR sequence, which can be distinguished from EPVS. **(F)** A 32-year-old male with cerebral cleft malformation combined with hypoplasia of the corpus callosum in the left cerebral hemisphere, who had recurrent headaches for many years. Multiple tumefactive PVS (white arrow) were seen in the subcortical white matter areas, and the lesions were unchanged on follow-up reviews of brain MR.

PVS appears striped when the penetrating artery is parallel to the imaging plane and rounded or ovoid when perpendicular to the imaging plane (Figures 3A–F) (1, 49). PVS is typically <3 mm in diameter (1). The distribution of PVS is usually bilaterally symmetrical. Sometimes, high-resolution MRI can reveal microscopic structures within the PVS: a central vessel can be observed in the cystic space and is defined as the “vessel sign”, which could be possibly helpful for differentiating the spaces from lacunes (Figure 3B) (1, 49). VR spaces can show a solitary round cyst, multiple strips and lines, unilateral predominantly dense cysts, or even a spider web-like appearance on MRI (Figure 3C).

PVS are commonly located in the basal ganglia, the centrum semiovale, the subcortical white matter of brain convexity, and the brainstem, however, the space is rarely seen in the cerebellum (1, 49). Large PVS is often first seen around the penetrating artery in the precribrum even in young people.

Perivascular spaces of 1–2 mm appear in all age groups (49, 50). Mildly enlarged PVS can also be detected in routine MR examination in healthy individuals of all ages, however, the burden of EPVS becomes increasingly apparent with advancing age (50). PVS of >3 mm can be observed in approximately one-third of elderly people (50). Increased visibility of VRS in the BG and white matter is significantly associated with age, and the VRS burden in the BG is heavier in men than in women (50). PVS with a diameter >3 mm must be distinguished from small lacunes. Lacunes tend to be 3–15 mm in diameter with a hyperintense rim around the vesicular cavity on FLAIR sequence (Figures 3D,E) (1). Sometimes, PVS can be especially enlarged with a diameter larger than 15 mm, even with mass effect, which is named as giant PVS or tumefactive PVS (Figure 3F) (51).

### Type of PVS

VR spaces are traditionally classified into three types based on their location. Type I-basal ganglia type: PVS courses with the lenticulostriate artery entering the basal ganglia through the precribrum (52); This is the most prevalent type. Type II-Hemispheric type: PVS follows the course of the medullary arteries into the white matter of brain convexity and extends into the subcortical white matter (52). Type III-midbrain type (Figure 3A): PVS lies in the midbrain following the path of penetrating artery from the posterior cerebral artery.

Similarly, giant VR spaces or tumefactive VR Spaces can be categorized into the three types described above, but type II and type III are more common (53). Type II VR spaces rarely cause clinical symptoms, but when they are diffuse, they may mimic PD or dementia (53). They are also seen in patients with neurofibromatosis and neuropsychiatric retardation (54, 55). Due to its special location, Type III VR spaces are more likely to compress the middle cerebral aqueduct or third ventricle, causing hydrocephalus. In some cases, tumefactive PVS can also cause pulsatile tinnitus (56), homonymous quadrantanopia (57), paralysis of the extremities (58), and reversible focal dystonia (59). Unlike the small PVS, the brain parenchyma surrounding the giant PVS has an abnormal signal intensity in 32.3% of type II cases and 3.8% of type III cases (53). They are often misdiagnosed as other pathologic processes, especially cystic neoplasms (49).

A recent study described an enlarged perivascular space located in the anterior temporal lobe with unique neuroimaging features (60). It occured in association with the vascular loop of the middle cerebral artery (MCA) branch and often compressed the adjacent cortex, with edema around the lesion. The clinical presentation was very similar to that of a cystic tumor and was often misdiagnosed and surgically removed as such. The author suggested that it should be considered as a separate subtype of perivascular spaces, namely type IV perivascular spaces, and proposed the term “opercular perivascular cyst” as naming. This type of PVS exhibits the same internal signal characteristics as CSF in all MRI sequences, without contrast enhancement or magnetic susceptibility artifacts, and the brain parenchyma surrounding the lesion shows variable high signal on T2WI and FLAIR (60).

### Quantification of Perivascular Spaces

#### Visual Scoring

**Figure 4:**
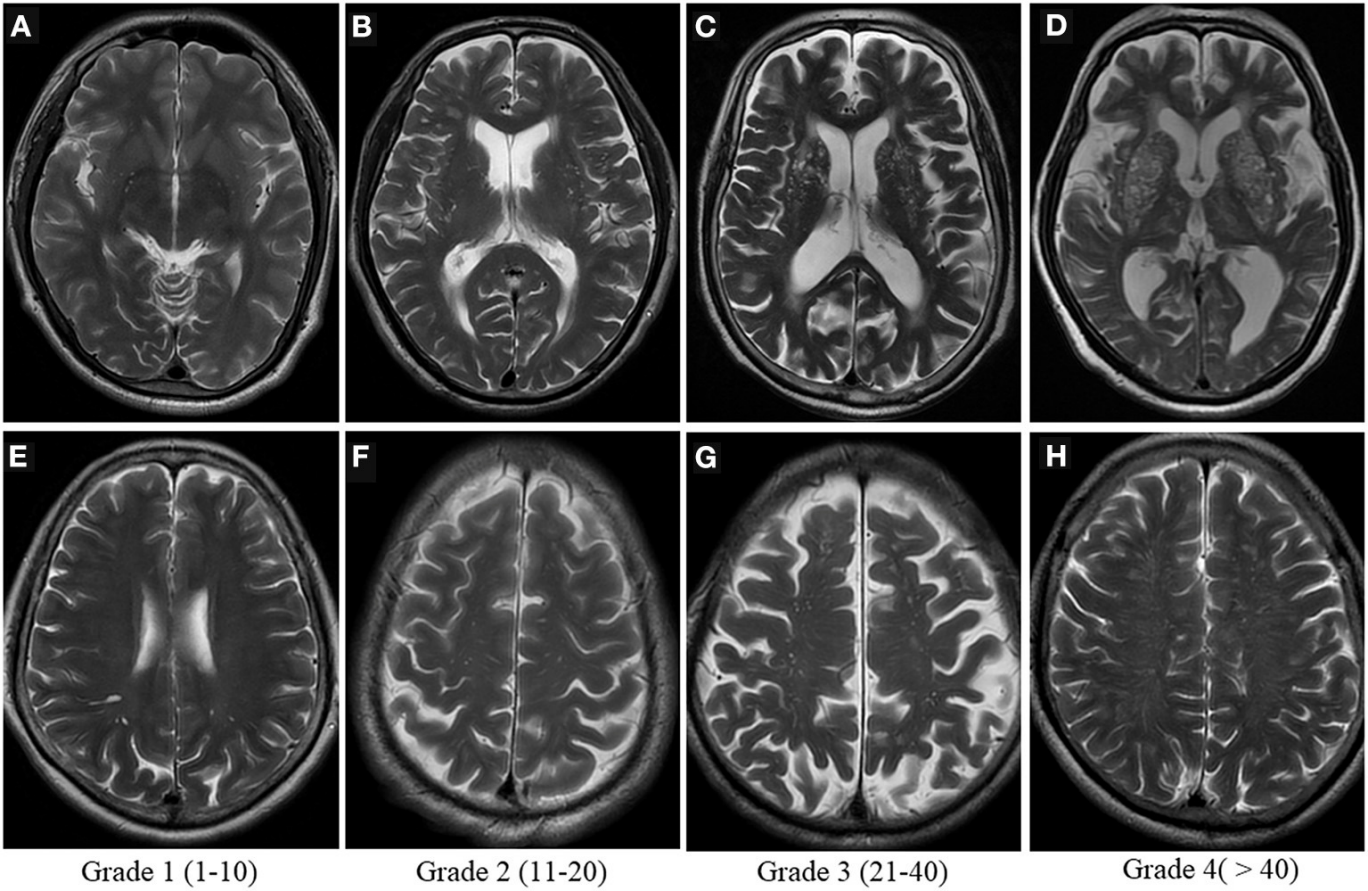
Visual scoring of PVS. Potter scoring-Axial T2WI demonstrates the different severity of EPVS in the basal ganglia [top row, **(A–D)**] and in the centrum semiovale [bottom row, **(E–H)**], with the corresponding scores (1–4) shown at the bottom.

Visual scoring is frequently used to assess the severity of PVS, but there is currently no uniform grading system for radiologists. The most widely used method is Potter scoring. PVS are divided into 0–4 grades (grade 0 none; grade 1 <1–10; grade 2 11–20; grade 3 21–40; grade 4 > 40) according to their number in the BG and centrum semiovale, by counting the side of the cerebral hemisphere with the heavier PVS burden, and 0 to 1 grades for the midbrain according to the presence or absence of PVS (Figure 4) (51). The hippocampus was not involved in the Potter scoring, which has numerous methods of grading. Chinese IntraCranial AtheroSclerosis (CICAS) study group graded the hippocampal region based on the number of PVS as follows: grade 1 <5; grade 2 5–10; grade 3 > 10 (29). Heier classified the PVS based on its diameter as: (mild) grade 1 <2 mm, (moderate) grade 2 2–3 mm, and (severe) grade 3 > 3 mm (52).

These scores are handy, practical, reliable and repeatable to use in clinical settings (51). Several studies, including some involving thousands of participants, have utilized these scoring methods (29). However, the intra- and inter-observer consistency of visual scores may be less than optimal, particularly in the centrum semiovale, as PVS coexists with other imaging markers of CSVD such as WMH and lacunae, making it difficult to distinguish between them (51). Visual scores are also subject to ceiling/flooring effects (3).

#### Automatic Computerized Quantification

With the development of computer image recognition technology, automatic computerized quantification of the perivascular space became feasible in clinical settings (61–63). Recently, Dubost et al. attempted to use 3D regression neural network for the quantification of dilated PVS, and the result was encouraging. The consistency between their automatic quantitative scoring and the experts' visual scores was high, with an intraclass correlation coefficient (ICC) of 0.74 and the reproducibility of the neural network was higher than the intra-rater agreement (ICC 0.93 vs. 0.80) (63). In addition, computational PVS counts correlate with age in a similar way to visual scores (63). Lian et al. presented a novel fully convolutional neural network to segment PVS efficiently without requiring any hand-drawing features or regions of interest (ROIs) (64). In addition to number, several morphological characteristics of perivascular spaces were also analyzed in some studies, including volume, size, length, width and linearity (65, 66). Interestingly, these studies showed that computational PVS morphological features were strongly associated with cerebrovascular risk factors and CSVD than PVS count or visual score (65, 66). The size and width of PVS in the centrum semiovale and deep corona radiata were associated with hypertension and stroke, but not with diabetes, hypercholesterolemia, or cardiovascular disease, while computational descriptors of PVS were associated with WMH severity in white matter region and with lacunes and microbleeds in basal ganglia (66).

Most of these automatic quantification methods focus on using computer technology in an effort to improve the efficiency and accuracy of automatic image segmentation. PVS is still mainly quantified visually and is usually limited to large-aperture PVS, and little has been done to improve its visibility and contrast with surrounding tissue through image postprocessing (67). Sepehrband et al. described an enhanced PVS contrast (EPC) multimodal approach to intensify the visibility of the PVS on MRI. An EPC image was created by fusing T1- and T2-weighted images and removing “non-structured high frequency spatial noise” using a dedicated filtering algorithm. This technique can significantly improve the consuity of the PVS and is effectively used for subsequent automatic quantification of the PVS (67).

These computational quantification methods are 100% automatic and do not have any inter- or intra-rater variability. This makes them more suitable for longitudinal studies. It seems that these methods have shown high consistency between manual assessment and computerized counts, but their value requires further testing. We expect that this automatic segmentation technique will be applied in the clinic in the near future, similarly to the AI software for lung nodule assessment that is widely used in radiology today. It will provide a wealth of basic data on the evolution of perivascular spaces with age and disease.

#### Application of Ultra-High-Field MRI

It is likely, in the near future, that ultra-high-field strength MRI will have an increased clinical impact. Ultra-high-field strengths are expected to improve spatial resolution and image contrast, making it possible to visualize the internal structure of VR spaces on MR images. Using 7T MRI in the human brain, small penetrating vessels within the PVS of the BG and centrum semiovale were successfully detected (48). Additionally, 7T MRI has been used to assess the asymmetric distribution of PVS in epileptic patients' brains (68), the number and size of VRS in multiple sclerosis patients (69), and the quantitative description of PVS in young and middle-aged healthy individuals (70, 71). Ultra-high-field MRI may help in the precise localization of onset areas of neurological disorders (68). Clearly, it is an effective tool for the non-invasive assessment of CSVD. It can detect atheromatous plaques in lenticulostriate arteries (72), assess post-stroke revascularization (73) and measure cerebral microvessel velocity (74).

Nevertheless, ultra-high field MRI also has its limitations. Typically, increased spatial resolution entails an increase in sensitivity to motion. An object scanned may exhibit artifacts caused by physiological involuntary movements, such as respiration or heartbeat, or spontaneous movements, such as slight head movements. By utilizing motion correction procedures, this issue can be greatly alleviated (75). The transmit radiofrequency (RF) magnetic field (B1) inhomogeneity also increases, resulting in a lower signal-to-noise ratio (SNR), especially in the periphery of the brain (75). Peripheral SNR loss may significantly limit detection of the subcortical perivascular spaces. In addition, the radiofrequency specific absorption rate (SAR) is significantly increased, which raises questions about the safety of the examination and the more limited compatibility with medical implants and devices (75). Parallel RF transmission (pTx) technology is an effective and most commonly applied solution for mitigating B1 inhomogeneities (76). Due to advances in pTx techniques in ultra-high magnetic field environments, combined with optimization of MR pulse sequences, black blood imaging techniques, non-contrast enhanced MR angiography (MRA) and arterial spin labeling (ASL) perfusion are able to achieve isotropic sub-millimeter resolution, which is very useful for high-resolution neurovascular imaging studies (76). Using optimized high-resolution black-blood T1-weighted 3D turbo spin-echo with variable flip angles (T1w TSE-VFA) sequences at 3T and 7T, Ma et al. successfully characterized the morphology of tonsillar arteries in healthy volunteers (77). As compared to 3T ASL, 7T ASL has a significantly better SNR, thus enabling whole-brain high-resolution perfusion imaging (76). Furthermore, ASL-based four-dimensional MRA (4D MRA) has been developed with a significantly higher SNR than ASL-based perfusion. 4D MRA at 7T enables detailed characterization of vascular structures and dynamic flow patterns (76). Regarding the safety of the examination at 7T, a recently proposed safety guideline can be helpful in future clinical settings for healthcare professionals (78).

## Imaging of Brain Glymphatic System

In the following section we will briefly discuss imaging techniques for assessing the glymphatic system, most of which are in development.

### Two-Photon Imaging and Fluorescent Tracing

Early studies of the cerebral glymphatic system were mostly carried out using two-photon imaging and fluorescence tracers. Iliff et al. used this technique to visualize the glymphatic pathway for the first time in rodents and showed that Aβ, the causative peptide of AD, was cleared from this pathway (5). The system is more effective during sleep (12, 14). A recent study explored the mechanisms of brain edema formation after stroke using radiolabeled tracers and multiphoton imaging coupled with DWI-MRI. According to the study, post-stroke cerebral edema is caused by an accelerated influx of CSF into the brain parenchyma via perivascular spaces within minutes of an ischemic insult (79). The finding overturns the traditional understanding of poststroke edema and provides a new concept for the development of alternative treatment strategies.

Researchers are continually innovating existing photo-microscopy techniques in order to obtain more accessible, minimally invasive, and higher definition images. Using a multiband filter cube, tunable LED light source, and CMOS camera, Sweeney et al. presented a transcranial fluorescence macroscopic imaging method to visualize CSF transport within perivascular spaces through the intact skull of living mice (80). In addition, a novel method for qualitative and quantitative estimation of glymphatic flux in rats using Evans blue-labeled albumin (EBA) was proposed that does not require radioactive labeling (81).

However, due to its drawbacks such as narrow field of view, shallow penetration depth, invasiveness to the study subject and the need for small cranial openings, this technique is not applicable to human brains. A real-time image of the entire human brain is not possible. Human brain researches are mainly focused on postmortem tissue assessment. Researchers evaluated the postmortem frontal cortex using Western blotting and immune-fluorescence from a total of 79 cognitively competent and histo-pathologically proven AD patients, and found a significant association between AQP4 expression and advancing age in all the participants (82). Individuals with AD had reduced AQP4 perivascular localization, and loss of perivascular localization slowed the clearance of Aβ and promoted its deposition (82). This may be one of the most successful applications of microscopy techniques in the human brain glymphatic system.

### MRI for Assessment of the Glymphatic System

MRI's sensitive, non-invasive nature has made it possible to bring the study of the glymphatic system from the laboratory to the clinic. Researchers today are becoming more and more interested in the application of novel MR imaging techniques for assessing the human glymphatic system.

#### Dynamic Contrast-Enhanced MRI, DCE-MRI

Similar to animal experiments, non-invasive direct *in vivo* imaging of the human glymphatic system has been mainly accomplished through intrathecal administration of contrast agents. Intrathecal injection of gadolinium-based contrast agents (GBCA) has been considered safe at doses <1.0 mmol (83, 84). Serious neurotoxic complications were associated with significantly higher GBCA doses (84).

Researchers have also tried to assess the glymphatic system using the faster and more convenient intravenous GBCA method, thus avoiding the need for intrathecal injections. For a long time, it has been widely assumed that GBCA does not cross an intact BBB (85). However, recent studies questioned this dogma (85–87). Today, it is generally accepted that gadolinium, even with intact BBB and competent renal function, can gradually accumulate in the human CSF after intravenous injection. Based on this consideration, one study utilized heavy T2-weighted fluid-attenuated inversion recovery imaging (hT2W-FLAIR), a technique they call delayed T2-weighted gadolinium enhancement imaging, to assess GBCA distribution in the brain at different time points after intravenous administration in a cohort of 33 healthy subjects and 7 patients with brain metastases, and confirmed that hT2W-FLAIR allows the observation of the pathway of GBCA into and out of the glymphatic system (88). There are at least two routes were found for GBCA to enter the glymphatic system: through the blood-aqueous barrier of the ciliary body, as well as through the choroid plexus into the CSF circulation, from where GBCA was eventually removed along the PVS of penetrating arteries and the cranial perineural sheath (88). Further comparative analysis revealed no significant differences in signal intensity at any of the observed locations between patients with brain metastases and subjects without neurological disease, while patients with severe deep and periventricular WMHs (Fazekas score = 3) had significantly higher signals in the PVS, aqueous chamber, choroid plexus and ventricles than patients with minimal white matter lesions (Fazekas score ≤ 1) (88). With MRI, Absinta and colleagues found a way to visualize lymphatic vessels in people and common marmoset monkeys' dura mater. High-resolution 3D cranial T1-Magnetization Prepared Rapid Acquisition of Gradient Echoes (T1-MPRAGE), limited T2-FLAIR, T1 black blood technique and T1-Sampling Perfection with Application optimized Contrasts using different flip angle Evolution (T1-SPACE) were applied to visualize human and primate meningeal lymphatic vessels by intravenous injection of two chemically different contrast agents, gadobutrol and gadofosveset (89). Gadobutrol is a macrocyclic contrast agent with a high propensity for extravasation and gadofosveset is a blood-pool agent. The results show that lymphatic vessels enhance with gadobutrol, but not with gadofosveset (89). Their achievement is an important step toward noninvasive visualization of human dural lymphatic vessels, and it offers hope for improving our knowledge of CNS' glymphatic drainage physiology. If this result can be repeatedly validated in future studies, in combination with suitable MR sequences, gadobutrol may prove excellent for the examination of downstream drainage channels in the human glymphatic system. Would it also be possible to observe the perivascular spaces with the appropriate adjustments to the experimental design?

#### Diffusion Imaging

DCE-MRI for the human glymphatic system has its own drawbacks (90): (1) the potential adverse effects of GBCA accumulation in the brain, and (2) the long time required for the GBCA to reach and transit through the PVS after injection, which may limit its clinical utility. Furthermore, GBCA-free imaging of the glymphatic system is becoming increasingly popular. DWI allows for indirectly assessing the movement of intracranial interstitial extracellular spaces (iECS) (90). Diffusion tensor image analysis along the perivascular space (DTI-ALPS) was applied to assess the glymphatic activity in AD cases. Impaired water diffusivity along PVS in the white matter with dense projection or association fibers was associated with lower mini-mental state examination (MMSE) score and AD severity (91). Similar results have been observed in Parkinson's patients (92). Another study in a community-dwelling population of non-demented older adults showed that a higher DTI-ALPS index was associated with higher scores on the neuropsychological performance test and also with higher gray matter volume (93). Meanwhile, the significant association between glymphatic function and sleep was reconfirmed (93). These studies suggest the promise of the DTI-ALPS index as an imaging marker for assessing cognitive impairment. Two other researches on sleep disorders found that the DTI-ALPS index was significantly lower in patients with obstructive sleep apnea (OSA) and isolated rapid eye movement sleep behavior disorder (iRBD) than in healthy controls (94, 95).

Intravoxel incoherent motion diffusion weighted imaging (IVIM-DWI) has also been extended to assess for glymphatic function. This noninvasive DWI technique is considered suitable for the measurement of both Brownian motion and microvascular perfusion of brain tissues. In the region surrounding EPVS and WMHs, an additional diffusion was detected independent of brain parenchymal diffusion and microvascular perfusion, using non-negative least squares IVIM-MRI spectral diffusion analysis (96). With increasing EPVS and WMH burden, the volume fraction of this additional intermediate diffusion increased (96).

#### Other Imaging Methods

As a novel MR perfusion imaging technique by using blood as its own tracer, instead of an exogenous contrast agent, arterial spin labeling (ASL) allows noninvasive assessment of cerebral perfusion (97). In terms of the brain glymphatic system, ASL was mainly used to evaluate BBB water permeability (97–99). A recent study utilized a diffusion prepared arterial spin labeling (DP-ASL) MRI approach to quantify the fluid exchange across the BBB [k(w)] among 40 cognitively normal older individuals, and found a positive correlation between BBB k(w) and CSF Aβ42 concentration levels in multiple brain regions (98). Current MRI techniques for detecting water exchange across BBB are summarized in a review paper by Dickie et al. and are divided into three categories. One is ASL-based methods mentioned above, including (1) Multi-TE ASL, (2) Diffusion-weighted ASL, (3) Magnetization transfer weighted ASL, (4) Contrast-enhanced ASL and (5) Phase- contrast ASL; second, contrast-based methods, including (1) Dose ramping at steady state with a varied infusion rate, (2) First pass methods, (3) Water exchange index (WEI) method and (4) Multiple flip angle multi-echo (MFAME)-MRI; third, methods based on the injection of MRI-detectable water tracers, including (1) Indirect detection of ^17^O-labeled water via its effect on ^1^H T_2_ and (2) Indirect detection of ^2^H-labeled water by proton replacement (100). These may be good alternative methods to study the aquaporin function at the level of capillaries. At the capillary level, the BBB and the glymphatic system have overlapping structural bases, and they may have synergistic and complementary roles in maintaining neuronal stability and the homeostasis of the intracerebral environment.

Chemical exchange saturation transfer (CEST) is a magnetic resonance spectroscopic imaging technique used to detect compounds at low concentrations, two orders of magnitude lower than standard magnetic resonance spectroscopy (MRS) imaging (90). By adding a saturation RF pulse to compounds with exchangeable hydrogen protons, the saturated hydrogen protons are transferred to the free water pool by chemical exchange, which results in a decrease in the free water signal. Information on compounds is thus obtained indirectly by detecting the free water signal changes. This emerging MRI technology can be used to detect body temperature, pH, enzyme activity and metabolites (glucose, glutamate, creatine and inositol) (101). In a porcine model of impaired glymphatic function, intrinsic compounds in the blood, lymph, and CSF were distinguished using CEST MRI at 7 Tesla, and the CEST effect of lymph is extremely significant compared to that of blood and CSF, which the author refers to as the “Lym-CEST” effect (102). In the subsequent animal experiment, this “Lym-CEST” effect was used to assess the impaired status of the glymphatic system in a rat model with unilateral cervical lymph node ligation. Results indicated that the ipsilateral hippocampus had a significantly higher signal intensity than the contralateral hippocampus, and this altered signal was strongly correlated with behavioral scores (102). According to these results, CEST MRI could be a useful tool to analyze metabolites and detect glymphatic function changes in the glymphatic system *in vivo*. To date, the application of CEST for the analysis of compounds within human brain lymph fluid has not been documented, but this does not prevent its great potential for exploring the glymphatic function and pathogenic mechanisms.

A combination of MRI and other imaging techniques can significantly improve sensitivity and accuracy, and provide a wide range of options for studying the glymphatic system. A combination of *in vivo* MRI and PET in a 4.2-year-old dog found that the pial-glial basement membranes were significantly thicker in the midbrain compared to other parts of the brain, indicating that CSF influx through perivascular pathway is most efficient in the midbrain (103). This finding was confirmed in a 12-year-old dog using a post-mortem electron microscopy study. This result offers a novel potential target for intrathecal drug delivery. It has been reported that MRI-guided Focused UltraSound (MRgFUS) can disrupt the local BBB integrity and facilitate gadobutrol contrast penetration into the brain parenchyma (104). The accumulation of gadobutrol in the PVS, subarachnoid space, and surrounding large draining veins has been detected in a few patients with AD or amyotrophic lateral sclerosis (ALS) using MRgFUS (104). Ultra-fast Magnetic Resonance EncephaloGraphy (MREG) is a less commonly used route to evaluate glymphatic pulsation mechanisms (105). With this technique, Kiviniemi et al. detected three types of physiological mechanisms that affect CSF pulsations: cardiac, respiratory, and very low frequency pulsations (105). According to another study, near-infrared spectroscopy (NIRS) can be used to study the water dynamics within the human brain, to monitor the brain function over time (including sleep period) and in conjunction with various magnetic neuroimaging techniques, particularly MREG (106). These researches provide novel approaches to noninvasively study glymphatic function *in vivo*.

## Conclusion and Outlook

Enlargement of the perivascular space is considered to be one of the early imaging signs of CSVD and neurodegenerative disease. With the advance in imaging techniques, its pathophysiological mechanisms have been studied in depth. What is known is that the PVS, as the anatomical basis of the glymphatic system, plays a crucial role in removing cerebral waste, participating in water circulation, and maintaining brain homeostasis. Knowledge of the structure, function, influencing factors and associated diseases of this system is also being constantly updated. MRI overcomes the shortcomings of microscopic imaging and provides noninvasive visualization of the whole glymphatic system, which holds great promise for the clinical study of the glymphatic system. It may be useful for the early diagnosis, monitoring, treatment and prognosis of diseases related to the glymphatic system.

In addition to providing an innovative theory of metabolite clearance, future studies of the glymphatic system will have to address many important questions, such as (1) the form of fluid exchange in the glymphatic system, the specific components that can enter the system and the mechanisms of entry, the specific types of waste products that can be removed, and whether iron excretion after cerebral hemorrhage also occurs through this conduit? (2) What are the exogenous regulatory approaches of the system? Research into the intrinsic mechanisms of operation, the factors involved, and how they are regulated needs to be further developed. (3) Its effects on CSF circulation need to be taken into account. The effects on non-neurological diseases need further study. For example, it has been tentatively demonstrated that the development of glaucoma is associated with dysfunction of the cerebral glymphatic system (107). (4) What is the utility of sleep therapy? (5) Can intrathecal drug delivery be an effective route of administration for CNS diseases? What is its safety profile? Can drugs that previously failed to cross the BBB by oral or intravenous routes be administered intrathecally, thus providing new perspectives for the development of new drugs. (6) Given the existence of the lymphatic system in CNS, why is it still rare for intracranial tumors to metastasis to the neck via this route and what are the reasons for this? (7) Longitudinal studies of perivascular spaces enlargement in large samples are still lacking. Is the perivascular spaces burden during childhood and youth associated with the development of AD or any other cognitive decline in the future? The study of the brain glymphatic system opens up new ideas for neurological disease research and brings new treatment strategies for patients, and we believe that with joint efforts, these questions will be overcome one by one.

## Author Contributions

All authors listed have made a substantial, direct, and intellectual contribution to the work and approved it for publication.

## Funding

This work was supported by Zhejiang Natural Science Foundation Association of Mathematical and Physical Medicine (No. LSY19H180014) and Zhejiang Basic Public Welfare Research Project (No. LGF19H220003).

## Conflict of Interest

The authors declare that the research was conducted in the absence of any commercial or financial relationships that could be construed as a potential conflict of interest.

## Publisher's Note

All claims expressed in this article are solely those of the authors and do not necessarily represent those of their affiliated organizations, or those of the publisher, the editors and the reviewers. Any product that may be evaluated in this article, or claim that may be made by its manufacturer, is not guaranteed or endorsed by the publisher.

## References

[1] Wardlaw JM Smith EE Biessels GJ Cordonnier C Fazekas F Frayne R. Neuroimaging standards for research into small vessel disease and its contribution to ageing and neurodegeneration. Lancet Neurol. (2013) 12:822–38. 10.1016/S1474-4422(13)70124-8 23867200 PMC3714437

[2] Nedergaard M. Neuroscience. Garbage truck of the brain. Science. (2013) 340:1529–30. 10.1126/science.1240514 23812703 PMC3749839

[3] Wardlaw JM Benveniste H Nedergaard M Zlokovic BV Mestre H Lee H. Perivascular spaces in the brain: anatomy, physiology and pathology. Nat Rev Neurol. (2020) 16:137–53. 10.1038/s41582-020-0312-z 32094487

[4] Agarwal N Carare RO. Cerebral Vessels: An overview of anatomy, physiology, and role in the drainage of fluids and solutes. Front Neurol. (2021) 11:611485. 10.3389/fneur.2020.611485 33519691 PMC7838613

[5] Iliff JJ Wang M Liao Y Plogg BA Peng W Gundersen GA. A paravascular pathway facilitates CSF flow through the brain parenchyma and the clearance of interstitial solutes, including amyloid β. Sci Transl Med. (2012) 4:147ra111. 10.1126/scitranslmed.3003748 22896675 PMC3551275

[6] Iliff JJ Lee H Yu M Feng T Logan J Nedergaard M. Brain-wide pathway for waste clearance captured by contrast-enhanced MRI. J Clin Invest. (2013) 123:1299–309. 10.1172/JCI67677 23434588 PMC3582150

[7] Louveau A Smirnov I Keyes TJ Eccles JD Rouhani SJ Peske JD. Structural and functional features of central nervous system lymphatic vessels. Nature. (2015) 523:337–41. 10.1038/nature14432 26030524 PMC4506234

[8] Jessen NA Munk AS Lundgaard I Nedergaard M. The Glymphatic System: a beginner's guide. Neurochem Res. (2015) 40:2583–99. 10.1007/s11064-015-1581-6 25947369 PMC4636982

[9] Shi Y Thrippleton MJ Blair GW Dickie DA Marshall I Hamilton I. Small vessel disease is associated with altered cerebrovascular pulsatility but not resting cerebral blood flow. J Cereb Blood Flow Metab. (2020) 40:85–99. 10.1177/0271678X18803956 30295558 PMC6928551

[10] Mestre H Tithof J Du T Song W Peng W Sweeney AM. Flow of cerebrospinal fluid is driven by arterial pulsations and is reduced in hypertension. Nat Commun. (2018) 9:4878. 10.1038/s41467-018-07318-3 30451853 PMC6242982

[11] Dreha-Kulaczewski S Joseph AA Merboldt KD Ludwig HC Gärtner J Frahm J. Identification of the upward movement of human CSF in vivo and its relation to the brain venous system. J Neurosci. (2017) 37:2395–402. 10.1523/JNEUROSCI.2754-16.2017 28137972 PMC6596847

[12] Xie L Kang H Xu Q Chen MJ Liao Y Thiyagarajan M. Sleep drives metabolite clearance from the adult brain. Science. (2013) 342:373–7. 10.1126/science.1241224 24136970 PMC3880190

[13] Berezuk C Ramirez J Gao F Scott CJ Huroy M Swartz RH. Virchow-robin spaces: correlations with polysomnography-derived sleep parameters. Sleep. (2015) 38:853–8. 10.5665/sleep.4726 26163465 PMC4434551

[14] Hablitz LM. PláV, Giannetto M, Vinitsky HS, Stæger FF, Metcalfe T, et al. Circadian control of brain glymphatic and lymphatic fluid flow. Nat Commun. (2020) 11:4411. 10.1038/s41467-020-18115-2 32879313 PMC7468152

[15] Mestre H Hablitz LM Xavier AL Feng W Zou W Pu T. Aquaporin-4-dependent glymphatic solute transport in the rodent brain. eLife. (2018) 7. 10.7554/eLife.40070 PMC6307855 30561329

[16] van Veluw SJ Hou SS Calvo-Rodriguez M Arbel-Ornath M Snyder AC Frosch MP. Vasomotion as a driving force for paravascular clearance in the awake mouse brain. Neuron. (2020) 105:549–61.e5. 10.1016/j.neuron.2019.10.033 31810839 PMC7028316

[17] Blair GW Thrippleton MJ Shi Y Hamilton I Stringer M Chappell F. Intracranial hemodynamic relationships in patients with cerebral small vessel disease. Neurology. (2020) 94:e2258–69. 10.1212/WNL.0000000000009483 32366534 PMC7357294

[18] Mestre H Kostrikov S Mehta RI Nedergaard M. Perivascular spaces, glymphatic dysfunction, and small vessel disease. Clin Sci (Lond). (2017) 131:2257–74. 10.1042/CS20160381 28798076 PMC5567781

[19] Smith AJ Yao X Dix JA Jin BJ Verkman AS. Test of the ‘glymphatic' hypothesis demonstrates diffusive and aquaporin-4-independent solute transport in rodent brain parenchyma. eLife. (2017) 6:e27679. 10.7554/eLife.27679 28826498 PMC5578736

[20] Benveniste H Liu X Koundal S Sanggaard S Lee H Wardlaw J. The glymphatic system and waste clearance with brain aging: a review. Gerontology. (2019) 65:106–19. 10.1159/000490349 29996134 PMC6329683

[21] Lundgaard I Lu ML Yang E Peng W Mestre H Hitomi E. Glymphatic clearance controls state-dependent changes in brain lactate concentration. J Cereb Blood Flow Metab. (2017) 37:2112–24. 10.1177/0271678X16661202 27481936 PMC5464705

[22] Achariyar TM Li B Peng W Verghese PB Shi Y McConnell E. Glymphatic distribution of CSF-derived apoE into brain is isoform specific and suppressed during sleep deprivation. Mol Neurodegener. (2016) 11:74. 10.1186/s13024-016-0138-8 27931262 PMC5146863

[23] Rangroo Thrane V Thrane AS Plog BA Thiyagarajan M Iliff JJ Deane R. Paravascular microcirculation facilitates rapid lipid transport and astrocyte signaling in the brain. Sci Rep. (2013) 3:2582. 10.1038/srep02582 24002448 PMC3761080

[24] Brown R Benveniste H Black SE Charpak S Dichgans M Joutel A. Understanding the role of the perivascular space in cerebral small vessel disease. Cardiovasc Res. (2018) 114:1462–73. 10.1093/cvr/cvy113 29726891 PMC6455920

[25] Li Y Li M Yang L Qin W Yang S Yuan J. The relationship between blood-brain barrier permeability and enlarged perivascular spaces: a cross-sectional study. Clin Interv aging. (2019) 14:871–78. 10.2147/CIA.S204269 31190773 PMC6519012

[26] Pettersen JA Keith J Gao F Spence JD Black SE. CADASIL accelerated by acute hypotension: Arterial and venous contribution to leukoaraiosis. Neurology. (2017) 88:1077–80. 10.1212/WNL.0000000000003717 28202707 PMC5384836

[27] Hladky SB Barrand MA. The glymphatic hypothesis: the theory and the evidence. Fluids Barriers CNS. (2022) 19:9. 10.1186/s12987-021-00282-z 35115036 PMC8815211

[28] Doubal FN MacLullich AM Ferguson KJ Dennis MS Wardlaw JM. Enlarged perivascular spaces on MRI are a feature of cerebral small vessel disease. Stroke. (2010) 41:450–4. 10.1161/STROKEAHA.109.564914 20056930

[29] Zhang C Chen Q Wang Y Zhao X Wang C Liu L. Chinese IntraCranial AtheroSclerosis (CICAS) Study Group. Risk factors of dilated Virchow-Robin spaces are different in various brain regions. PLoS ONE. (2014) 9:e105505. 10.1371/journal.pone.0105505 25157843 PMC4144854

[30] Charidimou A Hong YT Jäger HR Fox Z Aigbirhio FI Fryer TD. White matter perivascular spaces on magnetic resonance imaging: marker of cerebrovascular amyloid burden? Stroke. (2015) 46:1707–9. 10.1161/STROKEAHA.115.009090 25908461

[31] Charidimou A Meegahage R Fox Z Peeters A Vandermeeren Y Laloux P. Enlarged perivascular spaces as a marker of underlying arteriopathy in intracerebral haemorrhage: a multicentre MRI cohort study. J Neurol Neurosurg Psychiatry. (2013) 84:624–9. 10.1136/jnnp-2012-304434 23412074 PMC3905629

[32] Boulouis G Charidimou A Pasi M Roongpiboonsopit D Xiong L Auriel E. Hemorrhage recurrence risk factors in cerebral amyloid angiopathy: comparative analysis of the overall small vessel disease severity score versus individual neuroimaging markers. J Neurol Sci. (2017) 380:64–7. 10.1016/j.jns.2017.07.015 28870591 PMC5678990

[33] Hackett ML Pickles K. Part I: frequency of depression after stroke: an updated systematic review and meta-analysis of observational studies. Int J Stroke. (2014) 9:1017–25. 10.1111/ijs.12357 25117911

[34] Liang Y Chan YL Deng M Chen YK Mok V Wang F. Enlarged perivascular spaces in the centrum semiovale are associated with poststroke depression: a 3-month prospective study. J Affect Disord. (2018) 228:166–72. 10.1016/j.jad.2017.11.080 29253682

[35] Arba F Quinn TJ Hankey GJ Lees KR Wardlaw JM Ali M. Enlarged perivascular spaces and cognitive impairment after stroke and transient ischemic attack. Int J Stroke. (2018) 13:47–56. 10.1177/1747493016666091 27543501

[36] Hurford R Charidimou A Fox Z Cipolotti L Jager R Werring DJ. MRI-visible perivascular spaces: relationship to cognition and small vessel disease MRI markers in ischaemic stroke and TIA. J Neurol Neurosurg Psychiatry. (2014)85:522–5. 10.1136/jnnp-2013-305815 24249785 PMC3995332

[37] Smeijer D Ikram MK Hilal S. Enlarged Perivascular Spaces and Dementia: A Systematic Review. J Alzheimers Dis. (2019) 72:247–56. 10.3233/JAD-190527 31561362 PMC6839481

[38] Benjamin P Trippier S Lawrence AJ Lambert C Zeestraten E Williams OA. Lacunar infarcts, but not perivascular spaces, are predictors of cognitive decline in cerebral small-vessel disease. Stroke. (2018) 49:586–93. 10.1161/STROKEAHA.117.017526 29438074 PMC5832012

[39] Zafeiriou DI Batzios SP. Brain and spinal MR imaging findings in mucopolysaccharidoses: a review. AJNR Am J Neuroradiol. (2013)34:5–13. 10.3174/ajnr.A2832 22790241 PMC7966323

[40] Zeegers M Van Der Grond J Durston S Nievelstein RJ Witkamp T. Van Daalen E,et al. Radiological findings in autistic and developmentally delayed children. Brain Dev. (2006) 28:495–9. 10.1016/j.braindev.2006.02.006 16616445

[41] Ota Y Srinivasan A Capizzano AA Bapuraj JR Kim J Kurokawa R. Central nervous system systemic lupus erythematosus: pathophysiologic, clinical, and imaging features. Radiographics. (2022) 42:212–32. 10.1148/rg.210045 34990324

[42] Miyata M Kakeda S Iwata S Nakayamada S Ide S Watanabe K. Enlarged perivascular spaces are associated with the disease activity in systemic lupus erythematosus. Sci Rep. (2017) 7:12566. 10.1038/s41598-017-12966-4 28974720 PMC5626765

[43] Campi A Benndorf G Filippi M Reganati P Martinelli V Terreni MR. Primary angiitis of the central nervous system: serial MRI of brain and spinal cord. Neuroradiology. (2001) 43:599–607. 10.1007/s002340100561 11548164

[44] Salvarani C Brown RD Jr Huston J 3rd Hunder GG. Prominent perivascular enhancement in primary central nervous system vasculitis. Clin Exp Rheumatol. (2008) 26(Suppl. 49):S111. 18799067

[45] Sarkis RA Mays M Isada C Ahmed M MRI. findings in cryptococcal meningitis of the non-HIV population. Neurologist. (2015) 19:40–5. 10.1097/NRL.0000000000000000 25607331

[46] Tan ZR Long XY Li GL Zhou JX Long L. Spectrum of neuroimaging findings in cryptococcal meningitis in immunocompetent patients in China - a series of 18 cases. J Neurol Sci. (2016) 368:132–7. 10.1016/j.jns.2016.06.069 27538616

[47] Wardlaw JM Brindle W Casado AM Shuler K Henderson M Thomas B. A systematic review of the utility of 15 versus 3 Tesla magnetic resonance brain imaging in clinical practice and research. Eur Radiol. (2012) 22:2295–303. 10.1007/s00330-012-2500-8 22684343

[48] Bouvy WH Biessels GJ Kuijf HJ Kappelle LJ Luijten PR Zwanenburg JJ. Visualization of perivascular spaces and perforating arteries with 7 T magnetic resonance imaging. Invest Rdiol. (2014) 49:307–13. 10.1097/RLI.0000000000000027 24473365

[49] Kwee RM Kwee TC. Virchow-robin spaces at MR imaging. Radiographics. (2007) 27:1071–86. 10.1148/rg.274065722 17620468

[50] Zhu YC Dufouil C Mazoyer B. SoumaréA, Ricolfi F, Tzourio C, et al. Frequency and location of dilated Virchow-Robin spaces in elderly people: a population-based 3D MR imaging study. AJNR Am J Neuroradiol. (2011) 32:709–13. 10.3174/ajnr.A2366 21349956 PMC7965873

[51] Potter GM Chappell FM Morris Z Wardlaw JM. Cerebral perivascular spaces visible on magnetic resonance imaging: development of a qualitative rating scale and its observer reliability. Cerebrovasc Dis. (2015) 39:224–31. 10.1159/000375153 25823458 PMC4386144

[52] Heier LA Bauer CJ Schwartz L Zimmerman RD Morgello S Deck MD. Large Virchow-Robin spaces: MR-clinical correlation. AJNR Am J Neuroradiol. (1989) 10:929–36. 2505536 PMC8335297

[53] Kwee RM Kwee TC. Tumefactive Virchow-Robin spaces. Eur J Radiol. (2019) 111:21–33. 10.1016/j.ejrad.2018.12.011 30691661

[54] Li P Zhao F Liu P. Multiple large dilated Virchow-Robin spaces in a 12-year-old with neurofibromatosis type 2. Pediatr Neurol. (2014) 51:856–7. 10.1016/j.pediatrneurol.2014.08.018 25439494

[55] Härtel C Bachmann S Bönnemann C Meinecke P Sperner J. Familial megalencephaly with dilated Virchow-Robin spaces in magnetic resonance imaging: an autosomal recessive trait? Clin Dysmorphol. (2005) 14:31–4. 10.1097/00019605-200501000-00007 15602091

[56] Donaldson C Chatha G Chandra RV Goldschlager T. Obstructive hydrocephalus secondary to enlarged Virchow-Robin spaces: a rare cause of pulsatile tinnitus. World neurosurg. (2017) 101:815.e1–e3. 10.1016/j.wneu.2017.02.119 28268131

[57] Rivet A Gauthier AS Chatain M Billon-Grand R Thines L Delbosc B. Giant tumefactive Virchow-Robin space: a rare cause of a homonymous quadrantanopia. J Neuroophthalmol. (2017) 37:75–6. 10.1097/WNO.0000000000000478 28059864

[58] Ayele B Zenebe G Mengesha A Teshale Y. Symptomatic giant Virchow-Robin spaces: a rare cause of spastic quadriparesis in 43-year-old ethiopian patient: a case report. Ethiop J Health Sci. (2020) 30:843–6. 10.4314/ejhs.v30i5.24 33911846 PMC8047273

[59] De Schlichting E Zaldivar-Jolissaint JF Castrioto A Reyns N Chabardès S. Reversible focal dystonia secondary to giant perivascular spaces. Stereotact Funct Neurosurg. (2020) 98:80–4. 10.1159/000505711 32050205

[60] McArdle DJT Lovell TJH Lekgabe E Gaillard F. Opercular perivascular cysts: a proposed new subtype of dilated perivascular spaces. Eur J Radiol. (2020) 124:108838. 10.1016/j.ejrad.2020.108838 31972365

[61] Ballerini L Lovreglio R Valdés Hernández MDC Ramirez J MacIntosh BJ Black SE. Perivascular spaces segmentation in brain MRI using optimal 3D filtering. Sci Rep. (2018) 8:2132. 10.1038/s41598-018-19781-5 29391404 PMC5794857

[62] González-Castro V Valdés Hernández MDC Chappell FM Armitage PA Makin S Wardlaw JM. Reliability of an automatic classifier for brain enlarged perivascular spaces burden and comparison with human performance. Clin Sci. (2017) 131:1465–81. 10.1042/CS20170051 28468952

[63] Dubost F Adams H Bortsova G Ikram MA Niessen W Vernooij M. 3D regression neural network for the quantification of enlarged perivascular spaces in brain MRI. Med Image Anal. (2019) 51:89–100. 10.1016/j.media.2018.10.008 30390514

[64] Lian C Zhang J Liu M Zong X Hung SC Lin W. Multi-channel multi-scale fully convolutional network for 3D perivascular spaces segmentation in 7T MR images. Med Image Anal. (2018) 46:106–17. 10.1016/j.media.2018.02.009 29518675 PMC6430123

[65] Wang S Huang P Zhang R Hong H Jiaerken Y Lian C. Quantity and morphology of perivascular spaces: associations with vascular risk factors and cerebral small vessel disease. J Magn Reson Imaging. (2021) 54:1326–36. 10.1002/jmri.27702 33998738

[66] Ballerini L Booth T Valdés Hernández MDC Wiseman S Lovreglio R Muñoz Maniega S. Computational quantification of brain perivascular space morphologies: associations with vascular risk factors and white matter hyperintensities. a study in the lothian birth cohort 1936. Neuroimage Clin. (2020) 25:102120. 10.1016/j.nicl.2019.102120 31887717 PMC6939098

[67] Sepehrband F Barisano G Sheikh-Bahaei N Cabeen RP Choupan J Law M. Image processing approaches to enhance perivascular space visibility and quantification using MRI. Sci Rep. (2019) 9:12351. 10.1038/s41598-019-48910-x 31451792 PMC6710285

[68] Feldman RE Rutland JW Fields MC Marcuse LV Pawha PS Delman BN. Quantification of perivascular spaces at 7T: a potential MRI biomarker for epilepsy. Seizure. (2018) 54:11. 10.1016/j.seizure.2017.11.004 29172093 PMC5909959

[69] Kilsdonk ID Steenwijk MD Pouwels PJ Zwanenburg JJ Visser F Luijten PR. Perivascular spaces in MS patients at 7 Tesla MRI: a marker of neurodegeneration? Mult Scler. (2015) 21:155–62. 10.1177/1352458514540358 25013150

[70] Zong X Lian C Jimenez J Yamashita K Shen D Lin W. Morphology of perivascular spaces and enclosed blood vessels in young to middle-aged healthy adults at 7T: dependences on age, brain region, and breathing gas. NeuroImage. (2020) 218:116978. 10.1016/j.neuroimage.2020.116978 32447015 PMC7485170

[71] Zong X Park SH Shen D Lin W. Visualization of perivascular spaces in the human brain at 7T: sequence optimization and morphology characterization. Neuroimage. (2016) 125:895–902. 10.1016/j.neuroimage.2015.10.078 26520772

[72] Kong Q Zhang Z Yang Q Fan Z Wang B An J. 7T TOF-MRA shows modulated orifices of lenticulostriate arteries associated with atherosclerotic plaques in patients with lacunar infarcts. Eur J Radiol. (2019) 118:271–6. 10.1016/j.ejrad.2019.07.032 31439254

[73] Suzuki T Natori T Sasaki M Miyazawa H Narumi S Ito K. Evaluating recanalization of relevant lenticulostriate arteries in acute ischemic stroke using high-resolution MRA at 7T. Int J Stroke. (2021) 16:1039–46. 10.1177/1747493019897868 31955704

[74] Kang CK Park CA Lee DS Lee YB Park CW Kim YB. Velocity measurement of microvessels using phase-contrast magnetic resonance angiography at 7 Tesla MRI. Magn Reson Med. (2016) 75:1640–6. 10.1002/mrm.25600 25980462

[75] Barisano G Law M Custer RM Toga AW Sepehrband F. Perivascular space imaging at ultrahigh field MR imaging. Magn Reson Imaging Clin N Am. (2021) 29:67–75. 10.1016/j.mric.2020.09.005 33237016 PMC7694884

[76] Shao X Yan L Ma SJ Wang K Wang DJJ. High-resolution neurovascular imaging at 7T: arterial spin labeling perfusion, 4-dimensional MR angiography, and black blood MR imaging. Magn Reson Imaging Clin N Am. (2021) 29:53–65. 10.1016/j.mric.2020.09.003 33237015 PMC7694883

[77] Ma SJ Sarabi MS Yan L Shao X Chen Y Yang Q. Characterization of lenticulostriate arteries with high resolution black-blood T1-weighted turbo spin echo with variable flip angles at 3 and 7 Tesla. Neuroimage. (2019) 199:184–93. 10.1016/j.neuroimage.2019.05.065 31158475 PMC6688958

[78] Barisano G Culo B Shellock FG Sepehrband F Martin K Stevens M. 7-Tesla MRI of the brain in a research subject with bilateral, total knee replacement implants: case report and proposed safety guidelines. Magn Reson Imaging. (2019) 57:313–6. 10.1016/j.mri.2018.11.016 30496792 PMC9154312

[79] Mestre H Du T Sweeney AM Liu G Samson AJ Peng W. Cerebrospinal fluid influx drives acute ischemic tissue swelling. Science. (2020) 367:eaax7171. 10.1126/science.aax7171 32001524 PMC7375109

[80] Sweeney AW Plá V Du T Liu G Sun Q Peng S. In vivo imaging of cerebrospinal fluid transport through the intact mouse skull using fluorescence macroscopy. J Vis Exp. (2019) 149:10.3791/59774. 10.3791/59774 31403617 PMC7001880

[81] Wolf MS Chen Y Simon DW Alexander H Ross M Gibson GA. Quantitative and qualitative assessment of glymphatic flux using Evans blue albumin. J Neurosci Methods. (2019) 311:436–41. 10.1016/j.jneumeth.2018.09.031 30292824 PMC6258322

[82] Zeppenfeld DM Simon M Haswell JD D'Abreo D Murchison C Quinn JF. Association of perivascular localization of aquaporin-4 with cognition and Alzheimer disease in aging brains. JAMA Neurol. (2017) 74:91–9. 10.1001/jamaneurol.2016.4370 27893874

[83] Edeklev CS Halvorsen M Løvland G Vatnehol SAS Gjertsen Ø Nedregaard B. Intrathecal use of gadobutrol for glymphatic MR imaging: prospective safety study of 100 patients. AJNR Am J Neuroradiol. (2019) 40:1257–64. 10.3174/ajnr.A6136 31320462 PMC7048483

[84] Patel M Atyani A Salameh JP McInnes M Chakraborty S. Safety of intrathecal administration of gadolinium-based contrast agents: a systematic review and meta-analysis. Radiology. (2020) 297:75–83. 10.1148/radiol.2020191373 32720867

[85] Rasschaert M Weller RO Schroeder JA Brochhausen C Idée IM. Retention of gadolinium in brain parenchyma: pathways for speciation, access, and distribution. a critical review. J Magn Reson Imaging. (2020) 52:1293–305. 10.1002/jmri.27124 32246802 PMC7687192

[86] Nehra AK McDonald RJ Bluhm AM Gunderson TM Murray DL Jannetto PJ. Accumulation of gadolinium in human cerebrospinal fluid after gadobutrol-enhanced MR imaging: a prospective observational cohort study. Radiology. (2018) 288:416–23. 10.1148/radiol.2018171105 29737947

[87] Berger F Kubik-Huch RA Niemann T Schmid HR Poetzsch M Froehlich JM. Gadolinium distribution in cerebrospinal fluid after administration of a gadolinium-based MR contrast agent in humans. Radiology. (2018) 288:703–9. 10.1148/radiol.2018171829 29737953

[88] Deike-Hofmann K Reuter J Haase R Paech D Gnirs R Bickelhaupt S. Glymphatic pathway of gadolinium-based contrast agents through the brain: overlooked and misinterpreted. Invest Radiol. (2019) 54:229–37. 10.1097/RLI.0000000000000533 30480554

[89] Absinta M Ha SK Nair G Sati P Luciano NJ Palisoc M. Human and nonhuman primate meninges harbor lymphatic vessels that can be visualized noninvasively by MRI. eLife. (2017) 6:e29738. 10.7554/eLife.29738 28971799 PMC5626482

[90] Klostranec JM Vucevic D Bhatia KD Kortman HGJ Krings T Murphy KP. Current concepts in intracranial interstitial fluid transport and the glymphatic system: part II-imaging techniques and clinical applications. Radiology. (2021) 301:516–32. 10.1148/radiol.2021204088 34698564

[91] Taoka T Masutani Y Kawai H Nakane T Matsuoka K Yasuno F. Evaluation of glymphatic system activity with the diffusion MR technique: diffusion tensor image analysis along the perivascular space (DTI-ALPS) in Alzheimer's disease cases. Jpn J Radiol. (2017) 35:172–8. 10.1007/s11604-017-0617-z 28197821

[92] Ma X Li S Li C Wang R Chen M Chen H. Diffusion tensor imaging along the perivascular space index in different stages of Parkinson's disease. Front Aging Neurosci. (2021) 13:773951. 10.3389/fnagi.2021.773951 34867300 PMC8634754

[93] Siow TY Toh CH Hsu JL Liu GH Lee SH Chen NH. Association of sleep, neuropsychological performance, and gray matter volume with glymphatic function in community-dwelling older adults. Neurology. (2022) 98:e829mmuni 10.1212/WNL.0000000000013215 34906982

[94] Lee HJ Lee DA Shin KJ Park KM. Glymphatic system dysfunction in obstructive sleep apnea evidenced by DTI-ALPS. Sleep Med. (2022) 89:176–81. 10.1016/j.sleep.2021.12.013 35030357

[95] Lee DA Lee HJ Park KM. Glymphatic dysfunction in isolated REM sleep behavior disorder. Acta Neurol Scand. (2022) 145:464–70. 10.1111/ane.13573 34918348

[96] Wong SW Backes WH Drenthen GS Zhang CE Voorter PHM Staals J. Spectral diffusion analysis of intravoxel incoherent motion MRI in cerebral small vessel disease. J Magn Reson Imaging. (2020) 51:1170–80. 10.1002/jmri.26920 31486211 PMC7078988

[97] Joseph CR. Utilizing 3D arterial spin labeling to identify cerebrovascular leak and glymphatic obstruction in neurodegenerative disease. Diagnostics. (2021) 11:1888. 10.3390/diagnostics11101888 34679586 PMC8534509

[98] Gold BT Shao X Sudduth TL Jicha GA Wilcock DM Seago ER. Water exchange rate across the blood-brain barrier is associated with CSF amyloid-β 42 in healthy older adults. Alzheimers Dement. (2021). 10.1002/alz.12357 PMC8717840 33949773

[99] Joseph CR. Novel MRI techniques identifying vascular leak and paravascular flow reduction in early Alzheimer disease. Biomedicines. (2020) 8:228. 10.3390/biomedicines8070228 32698354 PMC7400582

[100] Dickie BR Parker GJM Parkes LM. Measuring water exchange across the blood-brain barrier using MRI. Prog Nucl Magn Reson Spectrosc. (2020) 116:19c.ate 10.1016/j.pnmrs.2019.09.002 32130957

[101] Tang Y Xiao G Shen Z Zhuang C Xie Y Zhang X. Noninvasive detection of extracellular pH in human benign and malignant liver tumors using CEST MRI. Front Oncol. (2020) 10:578985. 10.3389/fonc.2020.578985 33224880 PMC7667286

[102] Chen Y Dai Z Fan R Mikulis DJ Qiu J Shen Z. Glymphatic system visualized by chemical-exchange-saturation-transfer magnetic resonance imaging. ACS Chem Neurosci. (2020) 11:1978–84. 10.1021/acschemneuro.0c00222 32492333

[103] Dobson H Sharp MM Cumpsty R Criswell TP Wellman T Finucane C. The perivascular pathways for influx of cerebrospinal fluid are most efficient in the midbrain. Clin Sci. (2017) 131:2745–52. 10.1042/CS20171265 29021222

[104] Meng Y Abrahao A Heyn CC Bethune AJ Huang Y Pople CB. Glymphatics visualization after focused ultrasound-induced blood-brain barrier opening in humans. Ann Neurol. (2019) 86:975–80. 10.1002/ana.25604 31525269

[105] Kiviniemi V Wang X Korhonen V Keinänen T Tuovinen T Autio J. Ultra-fast magnetic resonance encephalography of physiological brain activity - Glymphatic pulsation mechanisms? J Cereb Blood Flow Metab. (2016) 36:1033–45. 10.1177/0271678X15622047 26690495 PMC4908626

[106] Myllylä T Harju M Korhonen V Bykov A Kiviniemi V Meglinski I. Assessment of the dynamics of human glymphatic system by near-infrared spectroscopy. J Biophotonics. (2018) 11:e201700123. 10.1002/jbio.201700123 28802090

[107] Wostyn P Killer HE De Deyn PP. Glymphatic stasis at the site of the lamina cribrosa as a potential mechanism underlying open-angle glaucoma. Clin Exp Ophthalmol. (2017) 45:539–47. 10.1111/ceo.12915 28129671

